# Identification and validation of the important role of tryptophan-related gene CYP1B1 in the development and progression of sepsis

**DOI:** 10.1371/journal.pone.0335944

**Published:** 2025-11-10

**Authors:** Li-Hong Chen, Wei-Ru Lin, Hui-Rong Hu, Zhi-Yuan Chen, Yan-Hui Wang

**Affiliations:** 1 Department of Anesthesiology, The Second Affiliated Hospital of Fujian Medical University, Quanzhou, China; 2 Department of Ultrasound, Quanzhou Maternity and Children’s Hospital, Quanzhou, Fujian, China; University of Michigan, UNITED STATES OF AMERICA

## Abstract

**Background:**

Tryptophan metabolism is involved in the progression and immune response of multiple diseases. However, the function of tryptophan metabolism in the immune characteristics of sepsis still needs to be elucidated.

**Methods:**

We collected the datasets from the GEO database to extract data of blood samples RNA of patients with or without sepsis in GSE28750, GSE65682, GSE69528, and GSE137342. A serials of bioinformatics analysis, including batch normalization, differential analysis, and single-cell sequencing analysis were finished through R software. Finally, the effects of the candidate differential gene on sepsis progression were evaluated using transcription-quantitative polymerase chain reaction (RT-qPCR), transwell assays, western blot, and immunofluorescence staining.

**Results:**

One tryptophan metabolism-related DEG for sepsis were obtained, namely CYP1B1. The transcriptional and translational level of CYP1B1 were obviously increased in the blood tissues. Notably, CYP1B1 exhibited great discriminative ability for the diagnosis of sepsis. Furthermore, single-cell sequencing analysis further indicated that CYP1B1 was primarily expressed in monocytes, and its’ expression level was significantly upregulated in monocytes activated by sepsis/LPS. Knockdown of CYP1B1 dramatically decreased the proinflammatory cytokines expression, blocked the migration of monocytes, and inhibited the expression of tryptophan metabolism-related protein TDO2.

**Conclusion:**

CYP1B1 is involved in tryptophan metabolism and its upregulation can promote the progression and development of sepsis through activating monocytes.

## Introduction

Sepsis is a life-threatening organ dysfunction caused by dysregulated host response to polymicrobial infections [1]. Its’ incidence increases rapidly in the recent years, leading to significant economic and societal costs. Thus, the World Health Organization (WHO) emphasizes sepsis as a worldwide healthcare priority [2,3]. Nevertheless, advances in intensive care techniques and antimicrobial therapy have been observed recently, but a large number of attempts regarding treatment for sepsis don’t generate inspiriting results [4]. This divergency majorly resulted from the complexity of pathological mechanism in sepsis. Thus, exploring and revealing the underlying pathogenesis and pathophysiological alterations of sepsis is extremely urgent.Tryptophan is an essential amino acid which is required for protein synthesis and severs as the precursor of several important compounds with crucial physiological functions [5,6]. Tryptophan and its metabolites play important roles in various physiological functions, including cell growth, regulation of the body’s response to the diet and environment, as well as generation of melatonin, 5-hydroxytryptamine (5-HT), and other bioactive molecules. There is increasing evidence that tryptophan metabolism disorders are closely involved in inflammatory and mental diseases [5–7]. It has been demonstrated that a large proportion of the biologically available tryptophan is broken down along kynurenine pathway, thus producing a series of intermediate metabolites that link to immune and inflammation response. In the recent years, the relationship between tryptophan and sepsis has been gradually revealed. For example, tryptophan exhaustion and nicotinamide adenine dinucleotide (NAD) accumulation caused by the activation of kynurenine pathway contributed to sustained immunological suppression in late stage of sepsis [8], which is one of the main reasons to cause fatal organ dysfunction in sepsis [9]. In addition, the level of plasma tryptophan was found to be decreased in septic patients, and the degree of reduction was associated with severity and prognosis of sepsis [10–12]. These findings indicated that tryptophan plays a crucial role in inflammatory processes and sepsis progression. Therefore, elucidating the molecular mechanisms behind tryptophan metabolism disorders has potential implications for therapy of sepsis.

In the present study, we comprehensively analyzed the Gene Expression Omnibus (GEO) database and the single-cell sequencing dataset in the sepsis dataset to depict the expression patterns of the tryptophan-related genes (TRPRGs) and validate these genes via in vitro experiments.

## Methods and material

### Collection and preprocessing of microarray data

We obtained four gene expression profile datasets associated with sepsis from GEO database. The datasets include: GSE28750 (GPL570), GSE65682 (GPL13667), GSE69528 (GPL10558), and GSE137342 (GPL10558), covering gene expression data from healthy volunteers and patients with sepsis. Detailed information of all downloaded datasets is summarized in Table 1. Initially, quality control and data preprocessing were performed using standard procedures to ensure data reliability and reproducibility. Raw expression data were first assessed for overall quality using boxplots to identify outlier samples. Normalization was carried out according to established methods described by Bolstad et al. [13] and Love et al. [14] to minimize technical variability and achieve comparable expression distributions across samples. To further address potential unwanted variation, including batch effects, we applied the ComBat algorithm implemented in the sva R package [15]. This empirical Bayes approach effectively adjusts for batch-associated biases while preserving biological variation. For additional validation, we examined the presence of residual batch effects using principal component analysis (PCA) before and after correction, following the recommendations of Leek et al. [16].

**Table 1:** Basic information of retrieved datasets.

GEO	Data type	Platform	Sample			Experiment Type	Attribute
			Total	Control	Sepsis		
GSE28750	mRNA	GPL570	30	20	10	array	training
GSE65682	mRNA	GPL13667	802	323	479	array	training
GSE69528	mRNA	GPL10558	138	55	83	array	training
GSE137342	mRNA	GPL10558	34	12	22	array	training
GSE69063	mRNA	GPL19983	46	17	29	array	Validation
GSE95233	mRNA	GPL570	90	22	68	array	Validation
GSE131761	mRNA	GPL13497	47	7	40	array	Validation

### Selection of differentially expressed genes

After merging the four datasets, differential analysis was performed using the Limma R package to screen differentially expressed genes (DEGs). |log2FC| > 0.5 and P < 0.05 were set as the screening criteria. Subsequently, volcano plot and heatmaps were drawn to visually present the expression characteristics of DEGs.

### Acquisition of hub TRPRGs (hTRPRGs) genes and construction of a PPI network

We obtained TRPRGs from the MSigDB (https://www.gsea-msigdb.org/gsea/msigdb) database [17], consisting of WP_TRY PTOPHAN_METABOLISM, KEGG_ TRYPT- OPHAN_METABOLISM, and REACTOME_TRYPTOPHAN_CATABOLISM. Subsequently, we identified hTRPRGs by intersecting DEGs and TRPRGs.

### Collection and processing of data for single-cell RNA-seq analysis

The single-cell RNA sequencing data from 7 samples of GSE167363 dataset (2 healthy volunteers and 5 sepsis samples) was used to determine the expression cells of hTRPRGs in peripheral blood mononuclear cells (PBMCs). The “Seurat” software package was used to analyze the single-cell sequencing data. Low-quality cells were removed by filtrating cells with expressed genes (>4000 and <200), genes expressed in less than 3 cells, the percent of mitochondrial genes >15%, the percent of hemoglobin genes <0.25%, and the percent of ribosome genes <60%. We then determined 20166 genes in a total of 33113 cells for further analysis. Subsequently, the batch effects were removed via the “Harmony” software package and the cell clusters were constructed using the “FindClusters” and “FindNeighbors” functions. The “TSNE” method was used to visualize these cell clusters and cellular annotation was performed based on the SingleR method. We utilized the “FindMarkers” function to analyze the DEGs between the two groups and their statistical significance was calculated using the Wilcoxon test (P < 0.05, LogFC > 1.5).

### Cell culture and transfection

Human monocyte lines THP-1 was purchased from the cell bank of the Chinese Academy of Sciences (Shanghai, China). THP-1 cells were cultured in RPMI 1640 medium (Gibco, ThermoFisher Scientific, United States) supplemented with 1% P/S (Gibco) and 10% fetal bovine serum at 37 °C with 5% CO_2_. Small interfering RNA (siRNA) targeting CYP1B1 and interfering RNA control were obtained from Gemma Genetics (Shanghai, China). For transient transfection, THP-1 cells were transfected with siRNA using a transfection reagent (Lipofectamine 2000) for 6 h, followed by expression validation and following experiments. This study only involves in vitro experiments, so an ethical statement is not required. In addition, Human Participants Research Checklist can be found in Supporting Information file 1.

### Real-time quantitative PCR (RT-qPCR)

We used RT-qPCR analysis to validate the expression level of hTRPRGs. Total RNA was isolated from THP-1 cells using TRIzol reagent (Thermo Fisher Scientific Inc., MA, USA) and was then reverse transcribed to generate cDNA using the PrimeScript™ RT reagent Kit (TaKaRa, Japan). RT-qPCR was conducted using TB Green™ Premix Ex Taq™ II (TaKaRa, Japan) and analyzed by Applied Biosystems software. The relative levels of each target gene were calculated via the 2^–ΔΔCt^ method.

The primer sequences were summarized in Table 2.

**Table 2:** mRNA-specific primers of genes.

Gene	Primer	Sequence (5’-3’)
GAPDH	FORWARD	GGCAAATTCAACGGCACAGTCAAG
	REVERSE	TCGCTCCTGGAAGATGGTGATGG
IL-1β	FORWARD	CACTACAGGCTCCGAGATGAACAAC
	REVERSE	TGTCGTTGCTTGGTTCTCCTTGTAC
IL-6	FORWARD	CTTCTTGGGACTGATGCTGGTGAC
	REVERSE	TCTGTTGGGAGTGGTATCCTCTGTG
TNFα	FORWARD	CGCTCTTCTGTCTACTGAACTTCGG
	REVERSE	GTGGTTTGTGAGTGTGAGGGTCTG
CYP1B1	FORWARD	AACGTACCGGCCACTATCAC
	REVERSE	TCACCCATACAAGGCAGACG

### Western blot assay

Total protein was isolated from THP-1 cells using RIPA lysis buffer (PC101; EpiZyme, Shanghai, China) and its’ concentration was determined using the bicinchoninic acid (BCA) method. Equal amounts of proteins (20 µg) were separated by 10% sodium dodecyl sulfate-polyacrylamide gel electrophoresis (SDS-PAGE) and transferred onto polyvinylidene fluoride (PVDF) membranes (IPVH00010; Millipore, Darmstadt, Germany). The membranes were blocked using 5% skim milk for 1 h at room temperature and then incubated with primary antibodies against CYP1B1 (A21196, 1:1000; ABclonal, Woburn, MA, USA), TDO2 (A18196, 1:1000; ABclonal), and GAPDH (AB2100, 1:7000; NCM, Newport, RI, USA) at 4 °C overnight. The membranes were incubated with secondary antibodies (G1213-100UL, 1:3000; Servicebio, Wuhan, China) at room temperature for 1 h. Finally, protein bands were analyzed and quantified using ImageJ software. The original WB blots can be found in Supporting Information file 2.

### Cell immunofluorescence

After being fixed with 4% paraformaldehyde for 1h at room temperature, cells were permeabilized with 0.2% Triton for 20 min and then blocked with 5% goat serum for 1h. Subsequently, cells were incubated with primary antibodies overnight for 14h. And then cells were incubated with fluorescein isothiocyanate (FITC) labeled goat anti-rabbit IgG (1:400, ZSGB-BIO) as secondary antibody at room temperature for 1 h. Afterward, the cells were treated with DAPI for 5 min. Imaging was obtained using an inverted Nikon confocal system (A1RS).

### Transwell assays

200 µl cell suspension (per well) were seeded into the upper chamber of a transwell plate and complete medium (500 µl) was added to the lower chamber of the transwell plate. After incubation for 24 h to allow cell migration, the cells were fixed with 4% paraformaldehyde for 30 min and then stained with 0.1% crystal violet for another 30 min. Microscopy images of the invaded cells were obtained, and these images were further analyzed using ImageJ software.

### Measurement of Kyn

Kynurenine (MEIMIAN) level in the supernatant of culture medium was determined according to manufacturer’s instructions. The mean optical density was detected at a wavelength of 450 nm and the content was calculated according to the standard curves and relative regression equations.

### Statistical analysis

R software (version 4.1.1) and GraphPad Prism 9 software were used to perform all statistical analyses. Student’s t-test (two groups) or one-way ANOVA (over two groups) was used to conduct Statistical evaluation of the data. All statistical analyses were two-sided and p < 0.05 indicated statistically significant

## Results

### Data processing and identification of hTRPRGs

**Fig 1:**
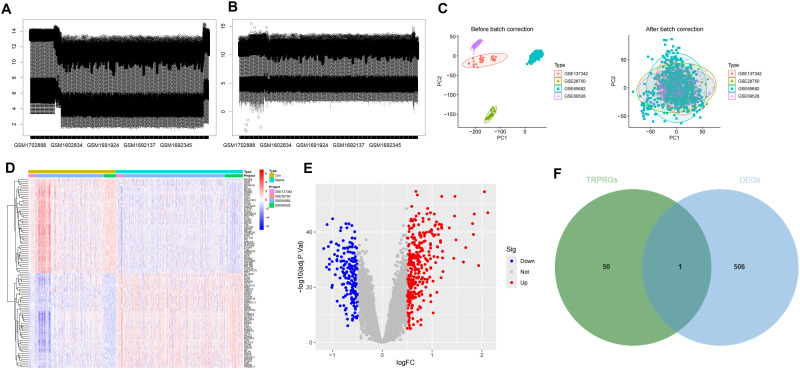
Differential expression analysis after merging datasets GSE28750, GSE65682, GSE69528, and GSE137342. (A) Box plot of gene expression before batch effect removal. (B) Box plot of gene expression after batch effect removal. (A) Box plot of gene expression before batch effect removal. (C) Principal component analysis (PCA) was employed to visualize batch correction efficacy before and after batch effect removal. (D) Heatmap showing significant differential expression of 40 genes. (E) Volcano plot of DEGs. (F) Venn plot.

First, GSE28750, GSE65682, GSE69528, and GSE137342 were merged into a cohort that included 410 healthy volunteers and 594 patients with sepsis. After removing batch effects, the data were normalized. The results of boxplot analysis (Fig 1A, 1B) (Supporting Information file 3) and PCA (Fig 1C) demonstrated the successful elimination of the batch effect. Differential analysis demonstrated a total of 507 DEGs, including 317 upregulated and 190 downregulated genes (Fig 1E) (Supporting Information file 4). Moreover, the top 100 DEGs were further displayed via a heatmap (Fig 1D). On the other hand, we obtained 51 TRPRGs from the MSigDB database. A total of 1 hTRPRG was both TRPRGs and DEGs: CYP1B1 (Fig 1F) (Supporting Information file 5).

### External validation of CYP1B1 expression

**Fig 2:**
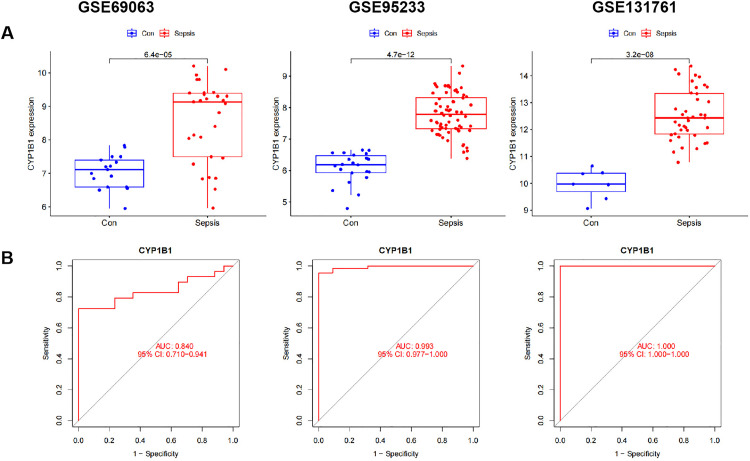
Identification of tryptophan metabolism-related gene CYP1B1 in sepsis. (A) The expression levels of CYP1B1. (B) ROC analysis for CYP1B1. ROC, receiver operator characteristic; AUC, area under the curve.

We verified the expression level of CYP1B1 using four external datasets (GSE69063, GSE95233, and GSE131761) (Supporting Information file 6) and found that CYP1B1 was significantly upregulated in sepsis group when compared with con group (Fig 2A, P < 0.05). Furthermore, receiver operating characteristic (ROC) curve analysis was conducted to assess the effectiveness of CYP1B1 for predicting sepsis. The AUC value was 0.840 (95% CI [0.710–0.941]) in the GSE69063 dataset, 0.993 (95% CI [0.977–1.000]) in the GSE95233 dataset, whereas it was equal to 1 in the GSE131761 dataset (Fig 2B). Overall, these outcomes indicated the strong predictive capability of CYP1B1 in exclusive diagnosis of patients with sepsis cases from healthy volunteers.

### Features related to CYP1B1 in the single-cell transcriptome

**Fig 3:**
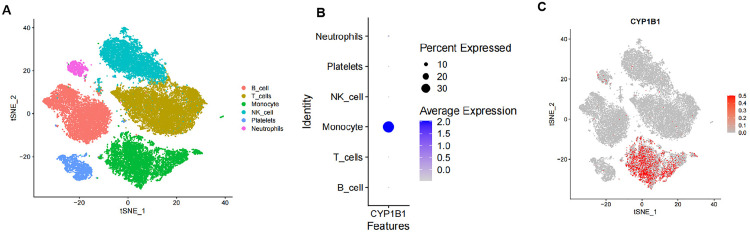
The specific expression of CYP1B1 were explored in different cell types at single-cell level. (A) After dimensionality reduction clustering of single cell data in GSE167363, a total of 6 different clusters were obtained. (B) The dotplot diagram showed the expression level of CYP1B1 in different cell types. (C) The UMAP graph showed the expression level of CYP1B1.

To further explore the specific cell type influenced by CYP1B1 in sepsis progression, single‐cell RNA‐seq was performed in blood samples from sepsis patients and healthy volunteers (GSE167363 dataset). After quality control and batch removal, we annotated cells into 6 major clusters, i.e., T cells, B cells, NK cells, monocyte, neutrophils, and platelets (Fig 3A) (Supporting Information file 7) using SingleR method. Importantly, analysis of single-cell sequencing results further showed that CYP1B1 was expressed in the monocytes (Fig 3B-C), indicating CYP1B1 probably involves in the development of sepsis by regulating the activity and function of monocytes.

### Expression validation of CYP1B1 mRNA and protein

**Fig 4:**
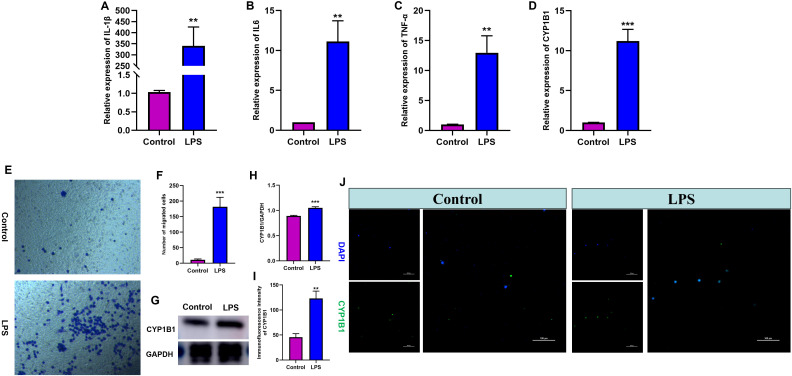
Validation of CYP1B1 expression in THP-1 monocytes. (A-D) RT-qPCR for the expressions of proinflammatory cytokines and CYP1B1. (E-F) Transwell assay of monocytes. (G-H) Western blot for the expression of CYP1B1. (I-J) Immuno-fluorescence staining for the expression of CYP1B1. **P < 0.01, ***P < 0.001.

In the aforementioned analysis, we confirmed a potential association of CYP1B1 with monocyte function and activity in sepsis, whereas that has not been comprehensively elucidated. Next, we firstly conducted a series of cell experiments, including RT-qPCR, western blot and immunofluorescence staining, to validate the expression change of CYP1B1 in THP-1 monocytes after LPS treatment. The results of RT-qPCR indicated that proinflammatory cytokines and CYP1B1 mRNA were significantly up-regulated in the LPS-activated THP-1 monocytes compared to control THP-1 cells (Fig 4A-D, P < 0.05). In addition, LPS stimulus obviously enhanced the migration ability of monocytes (Fig 4E-F, P < 0.05). As expected, the results from western blot and immunofluorescence staining further demonstrated that the expression level of CYP1B1 protein was obviously higher in the THP-1 cells activated by LPS, when compared with control THP-1 cells (Fig 4G-J, P < 0.05). These findings were consistent with those arising from external validation analysis, thus indicating that the data mining was reliable and the gene has valuable research value.

### CYP1B1 promotes monocyte activation and affects tryptophan metabolism

**Fig 5:**
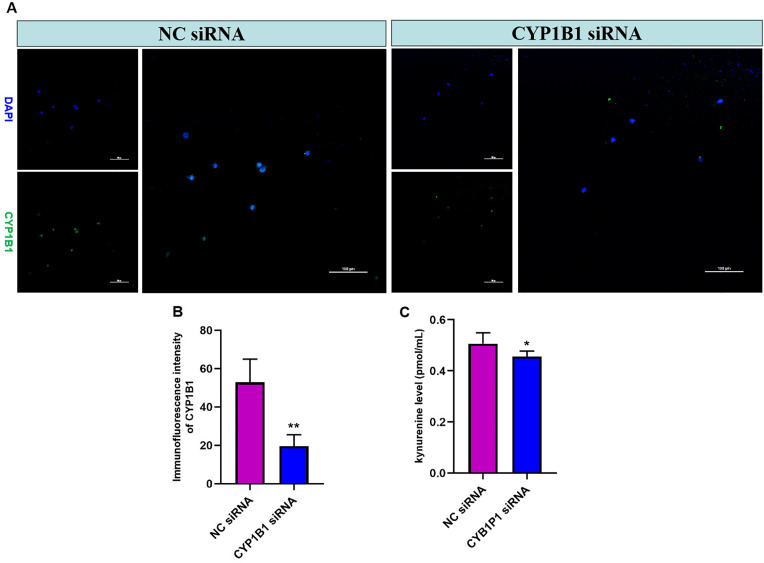
The transfection efficiency of CYP1B1 knockdown in THP-1 cells. *, P < 0.05, compared with NC siRNA group; **, P < 0.01, compared with NC siRNA group.

**Fig 6:**
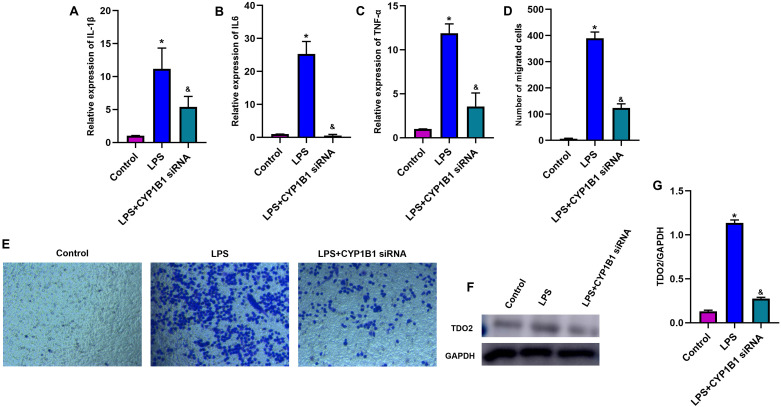
Knockdown of CYP1B1 inhibited monocytes activation and tryptophan metabolism. (A-C) RT-qPCR for the expressions of proinflammatory cytokines. (D-E) Transwell assay of monocytes. (F-G) Western blot for the expression of TDO2. *, P < 0.05, compared with Control group; &, P < 0.05, compared with LPS group.

In order to explore the role of CYP1B1 on monocytes, we knocked down CYP1B1 using siRNA. The transfection efficiency in THP-1 cells was investigated by confirming the protein expression level of CYP1B1 and the level of kynurenine, and it was revealed that CYP1B1 expression and kynurenine level were successfully inhibited in transfected cells compared with non-transfected NC cells (Fig 5, P < 0.05). Following this, the mRNA levels of proinflammatory cytokines in THP-1 cells were investigated using RT-qPCR, and the results indicated that following transfection, the levels of inflammatory factors expressed by LPS-activated THP-1 cells were obviously suppressed (Fig 6A-C, P < 0.05). Subsequently, the effect of CYP1B1 interference on the migration ability of THP-1 cells was investigated in this study by performing Transwell assays. The results revealed that compared with the LPS group, the migration rates exhibited by transfected THP-1 cells were obviously decreased (Fig 6D-E, P < 0.05). Importantly, western blot and Elisa results demonstrated that knockdown of CYP1B1 significantly downregulated the key tryptophan regulator (TDO2), demonstrating a potential fact that CYP1B1 involves in tryptophan metabolic processes (Fig 6F-G, P < 0.05).

## Discussion

Generally, sepsis is characterized by the dysregulation of host response to infectious pathogens [18]. The activation and facilitation of an inflammatory cascade is regarded as a crucial basis for the pathophysiologic shifts in sepsis [19]. It has been demonstrated that a variety of pathophysiologic mechanisms are related to septic organ injury, including coagulation disorders, endoplasmic reticulum stress, immunosuppression, dysregulated inflammation response [20]. Over the past decade, the development and improvement of diagnosis and treatment strategies have greatly prolonged the survival time of patients with sepsis. Nevertheless, the lack of effective molecular biomarkers hinders the timely diagnosis and therapy of septic patients. Thus, it’s urgent to elucidate the potential mechanism of sepsis to further reveal the precise molecular targets and medications for the treatment of sepsis.

Recently, tryptophan metabolism has aroused broad attention in the research field. Tryptophan is an important element for protein synthesis, and the metabolite of tryptophan is demonstrated to promote the activation of kynurenine associated pathway [21]. In tryptophan metabolism, TDO2 can promote the oxidization of tryptophan to produce formylkynurenine, and the latter is further degraded to kynurenine [22]. Thus, among tryptophan metabolites, kynurenine accounts for about 95% production of ingested tryptophan [23]. Previous literatures have confirmed the roles of tryptophan metabolism in sepsis. For instance, Wang et al. demonstrated that ozone modulates tryptophan metabolism and protects against sepsis-induced intestinal injury through activation of the aryl hydrocarbon receptor [24]. Similarly, Gao et al. reported that dysregulation of tryptophan metabolism contributes to cognitive impairment in a mouse model of sepsis-associated encephalopathy [25]. Additionally, elevated plasma kynurenine levels and increased kynurenine-to-tryptophan ratios following major trauma can also serve as early indicators of sepsis development [26]. Tryptophan catabolism through the kynurenine pathway directly modulates vascular tone, and promotes the development of hypotension in patients with septic shock [27]. However, the molecular mechanisms underlying the association between tryptophan metabolism and sepsis remain largely unclear. In this study, we characterized the molecular landscape of tryptophan metabolism in the context of sepsis and identified potential regulatory targets through systematic bioinformatics analysis, aiming to provide valuable insights and directions for future investigations into the pathogenesis of sepsis.

In this study, we first employed the public GEO database to validate DEGs between septic patients and healthy controls, and identified 183 upregulated and 61 downregulated genes from merged cohort of four datasets. Subsequently, these DEGs were imported into DAVID tool [28] to perform GO and KEGG enrichment analyses and found that these genes were enriched in biological processes (BP), cellular components (CC), and molecular functions (MF), revealing core biological pathways related to the development of sepsis. Afterward, we confirmed 1 hTRPRG (CYP1B1) through the intersection of the 507 DEGs and 51 TRPRGs. Lastly, we used another four datasets to validate the expression change and diagnostic performance of CYP1B1 in sepsis patients. Wilcoxon test demonstrated that CYP1B1 was obviously upregulated in sepsis samples, when compared with healthy samples. Meanwhile, ROC curve analysis further suggested that this gene shows good clinical diagnostic performance.

Cytochrome P450 1B1 (CYP1B1) is a member of Cytochrome P450 enzymes (CYP450s) that catalyzes NADPH-supported monooxygenation of various endogenous molecules and xenobiotics. CYP1B1 is generally induced by diverse proinflammatory and damaging substances, and primarily expressed in a variety of support cells, such as monocyte, endothelia, mesenchymal progenitor cells, pericytes, and stellate cells [29–31]. Previous literatures demonstrated that CYP1B1 is involved in carcinogenesis via the detoxication of exogenous aromatic hydrocarbons [32]. Additionally, CYP1B1 also increased the risk for breast cancer through promoting the production of reactive quinones that lead to DNA damage [33]. Notably, many literatures have validated a role for CYP1B1 in inflammation. For example, CYP1B1 deletion has been reported to diminish adult obesity and liver inflammation [34]. Furthermore, CYP1B1-deficient retinal astrocytes are protected from inflammation and oxidative stress [35]. However, the cellular and molecular mechanisms by which CYP1B1 impacts the development and progress of sepsis need further elucidation. In this study, we found that the expression of tryptophan metabolism-related CYP1B1 was significantly increased in blood samples of septic patients, indicating its potential as a hazardous gene in sepsis. In addition, CYP1B1 was also found to exhibit strong predictive capability in exclusive diagnosis of patients with sepsis cases from healthy volunteers.

Subsequently, we used sing-cell sequencing analysis to reveal a specific increase in CYP1B1 expression in monocytes. Notably, this result is crucial as monocytes are key players in the immune-inflammation response, contributing to sepsis progression and development [36,37]. However, the specific function of CYP1B1 expression in these immune cells remains to be further elucidated. The present study found that CYP1B1 and tryptophan metabolism-related molecule TDO2 expression was obviously increased in LPS-activated monocytes. Furthermore, CYP1B1 knockdown could effectively inhibit the monocytes activity, as evidenced by the reduced migrated rates and decreased inflammatory factors level. Importantly, CYP1B1 knockdown also significantly downregulated the expression of TDO2 and decreased the level of kynurenine. These findings indicated that targeting CYP1B1 probably induces the inhibition of tryptophan metabolism via blocking the expression of TDO2. This emphasized the potential of targeting CYP1B1 and tryptophan metabolism pathways as therapeutic strategies in sepsis. The therapies targeting modulation of CYP1B1 activity or tryptophan metabolism could influence the sepsis deterioration and improve patient prognosis.

However, several limitations of this study should be acknowledged. First, although ROC analysis indicates that the key target exhibits strong diagnostic performance, the validation datasets were also obtained from GEO. Inclusion of independent clinical samples will provide more robust evidence. Second, the experimental validation was limited to THP-1 cells stimulated with LPS. While this is a standard approach, it represents an artificial model, and the study will be substantially strengthened by using primary monocytes or patient-derived cells. Third, a direct causal relationship between alterations in tryptophan metabolism and the attenuation of the inflammatory phenotype has not been established. It remains unclear whether the observed anti-inflammatory effect is a direct consequence of CYP1B1 deficiency or is mediated via downregulation of the TDO2/kynurenine pathway. A rescue experiment—such as supplementing CYP1B1-knockdown cells with exogenous kynurenine to determine whether the inflammatory phenotype is restored—will considerably reinforce this claim

Overall, our study indicates that genes involved in tryptophan metabolism contribute to elucidating the molecular basis of sepsis and may offer novel perspectives for therapeutic intervention and immune regulation. Among these genes, CYP1B1 appears to enhance disease progression and holds promise as a biomarker for sepsis diagnosis and progression. Nevertheless, additional validation using larger, prospective patient cohorts is still needed.

## Supporting information

S1 File PLOSOne_Human_Subjects_Research_Checklist. (DOCX)

S2 File POriginal blots. (XLSX)

S3 File PTable S1 The gene expression before batch effect removal. (XLSX)

S4 File PTable S2 Differentially expressed genes. (XLSX)

S5 File PTable S3 The insection of tryptophan-related genes (TRPRGs) with differentially expressed genes (DEGs). (XLSX)

S6 File PTable S4 Expression validation of CYP1B1. (XLSX)

S7 File PTable S5 Markers of each cell cluster. (XLSX)
